# Successful Forceps Biopsy of Carcinoma of the Distal Bile Duct Under Direct Visualization Using a Stone Retrieval Balloon Catheter

**DOI:** 10.7759/cureus.48468

**Published:** 2023-11-07

**Authors:** Koichiro Mandai, Takato Inoue

**Affiliations:** 1 Department of Gastroenterology, Kyoto Second Red Cross Hospital, Kyoto, JPN

**Keywords:** distal, biopsy, bile duct, balloon, ampullary

## Abstract

We present the case of a 72-year-old woman with elevated hepatobiliary enzymes and a small mass in the distal bile duct near the papilla. Fluoroscopy-guided forceps biopsy initially yielded insufficient tissue. After endoscopic papillary large balloon dilation with sphincterotomy, a stone retrieval balloon was used to expose the tumor to the duodenum. Biopsy under direct visualization using standard forceps revealed adenocarcinoma. The technique may be useful for the biopsy of lesions located in the terminal segment of the distal bile duct.

## Introduction

A recent randomized controlled trial demonstrated the high diagnostic accuracy of cholangioscopy-guided biopsy for biliary diseases [1]. However, a previous study indicated that the presence of the lesion in the distal bile duct was a factor affecting a low tissue acquisition rate in cholangioscopy-guided forceps biopsy for non-stenotic bile ducts [2]. The limited flexion of the scope tip, making it difficult to target the desired biopsy site, and the difficulty in stabilizing the scope at the distal end of the bile duct are the possible reasons for this. Here, we present a successful forceps biopsy of distal bile duct carcinoma achieved through direct visualization using a stone retrieval balloon catheter.

## Technical report

**Figure 1:**
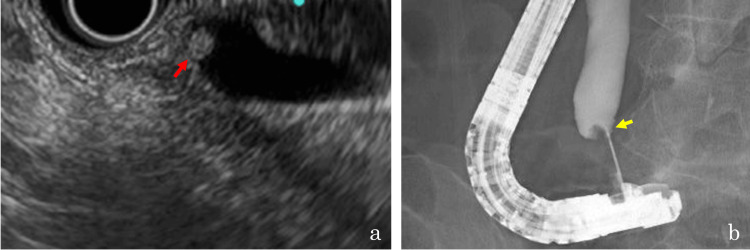
A polypoid lesion in the distal bile duct near the papilla. (a) Endoscopic ultrasonography shows a 7 mm low-echoic polypoid lesion (red arrow) in the distal bile duct near the papilla. (b) Fluoroscopy-guided forceps biopsy is performed for a polyp-like defect (yellow arrow) in the distal bile duct near the orifice of the papilla.

A 72-year-old woman was referred to our department with elevated levels of hepatobiliary enzymes. Contrast-enhanced computed tomography revealed a 5 mm mass in the distal bile duct near the papilla, which was recognized as a 7 mm low-echoic polypoid lesion upon endoscopic ultrasonography (Figure 1a). Endoscopic retrograde cholangiography (ERC) revealed an 8.0 × 4.7 mm polyp-like defect in the distal bile duct near the papillary orifice. Using intraductal ultrasonography with a 20 MHz mini-probe, the lesion was identified as a hypoechoic 4.4 mm mass. A biopsy forceps was advanced into the distal bile duct, and a fluoroscopy-guided forceps biopsy was performed (Figure 1b); however, sufficient tissue was not obtained.

**Figure 2:**
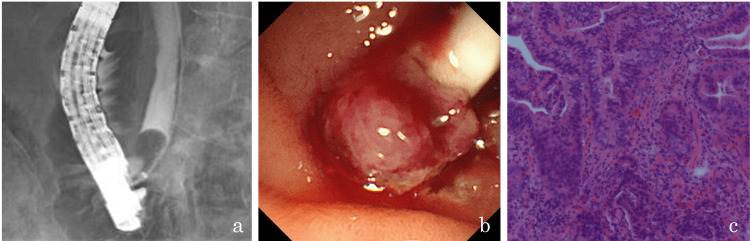
Exposing the tumor to the duodenum with a stone retrieval balloon catheter. (a) Fluoroscopy demonstrates that the lesion is pulled toward the duodenum using a stone retrieval balloon. (b) Part of the tumor with irregular mucosa and dilated vessels is exposed to the duodenum. (c) Adenocarcinoma is diagnosed via histopathological analysis with hematoxylin and eosin staining.

Bile cytology and biopsy tested negative for malignancy; therefore, ERC was repeated. Similar to the extraction of bile duct stones using a balloon catheter, to expose the lesion to the duodenum, endoscopic papillary large balloon dilation with sphincterotomy was initially performed. Subsequently, a balloon catheter was inserted into the bile duct. The balloon was inflated and pulled toward the duodenum in an attempt to expose the lesion to the duodenum (Figure 2a). Part of the tumor with irregular mucosa and dilated vessels was exposed to the duodenum (Figure 2b) and an endoscopic biopsy using standard biopsy forceps was performed. Biopsy results indicated adenocarcinoma (Figure 2c); hence, subtotal stomach-preserving pancreaticoduodenectomy was performed.

The final diagnosis was ampullary region carcinoma limited to the mucosa of the terminal segment of the distal bile duct.

## Discussion

In the pathological examination of biliary diseases, various methods, such as bile cytology, brush cytology, fluoroscopy-guided biopsy, cholangioscopy-guided biopsy, and endoscopic ultrasound-guided tissue acquisition (EUS-TA), are available [1-4]. A systematic review and meta-analysis reported pooled sensitivities of 45% for brush cytology and 48.1% for fluoroscopy-guided forceps biopsy [3]. In this case, as a bile duct stricture was not evident, brush cytology was omitted, and the small size of the lesion resulted in insufficient tissue from fluoroscopy-guided forceps biopsy.

For further diagnosis, cholangioscopy-guided forceps biopsy was considered. A recent randomized controlled trial demonstrated significantly higher diagnostic accuracy for cholangioscopy-guided biopsy compared to brush cytology (87.1% vs. 65.5%, respectively) [1]. However, tissue acquisition via cholangioscopy-guided forceps biopsy is often challenging for the tumor located in the terminal segment of the distal bile duct [2]. In this case, cholangioscopy-guided forceps biopsy was deemed challenging due to the small size and location of the tumor.

Although the diagnostic accuracy of EUS-TA for biliary diseases is reported as 87% [4], it may not be applicable in cases where puncturing masses while avoiding the biliary tract lumen is unattainable. In this case, EUS-TA was considered inapplicable due to the small size of the lesion confined within the biliary tract lumen.

Although our described technique does not guarantee that the tumor in the lower bile duct will always be visible from the duodenum, it is considered an acceptable approach when the tumor cannot be diagnosed through other methods or when the implementation of other methods is challenging.

## Conclusions

In summary, we present the case of a 72-year-old woman with a small carcinoma of the distal bile duct, successfully diagnosed through forceps biopsy under direct visualization using a stone retrieval balloon catheter. Our technique may be useful for the biopsy of lesions located in the distal bile duct near the papilla.
